# Hand grip strength and cognitive dysfunction amongst older Africans in Nigeria

**DOI:** 10.1371/journal.pone.0342598

**Published:** 2026-02-27

**Authors:** Rufus O. Akinyemi, Oladotun Victor Olalusi, Gabriel Ogunde, Tolulope Akinyemi, Joseph Yaria, Olabode Oguntiloye, Ayotomiwa Fagbemi, Eniola Cadmus, Femi Popoola, Mayowa Ogunronbi, Dorcas Olujobi, Olaoluwa Famuyiwa, Joshua Akinyemi, Mayowa Owolabi, Roman Romero-Ortuno, Adesola Ogunniyi, Raj Kalaria, Brian Lawlor

**Affiliations:** 1 Neuroscience and Aging Research Unit, Institute of Advanced Medical Research and Training, College of Medicine, University of Ibadan, Ibadan, Nigeria; 2 Department of Neurology, University College Hospital, Ibadan, Oyo, Nigeria; 3 College of Medicine, University of Ibadan, Ibadan, Oyo, Nigeria; 4 Lead City University, Ibadan, Oyo, Nigeria; 5 Department of Epidemiology and Medical Statistics, College of Medicine, University of Ibadan, Ibadan, Nigeria; 6 Global Brain Health Institute, Trinity College, Dublin, Ireland; 7 Africa Dementia Consortium, Ibadan, Ibadan, Nigeria; 8 Translational and Clinical Research Institute, Newcastle University, Campus for Ageing and Vitality, Newcastle Upon Tyne, United Kingdom; 9 Global Brain Health Institute, Trinity College Dublin, Dublin, Ireland; 10 Trinity College Institute of Neuroscience, School of Psychology, Trinity College Dublin, Dublin, Ireland; 11 St James Hospital, Dublin, Ireland; Ritsumeikan University, JAPAN

## Abstract

**Background:**

The relationship between physical and cognitive health among Africans, known for a rising incidence of frailty, cardiometabolic, and cognitive disorders, is unclear. We investigated the relationship between hand grip strength (HGS), and cognitive impairment among older adults in an urban settlement in Ibadan, South West Nigeria.

**Methods:**

In this study, we assessed 608 participants from the Vascular heAlth, fraiLty, and cognItion in Ageing Nigerians sTudy [VALIANT] – a population-based cohort of 1021 older persons in Ibadan, a city in Southwestern Nigeria. They were recruited through a multi-stage, stratified cluster random sampling method. Data on HGS were obtained using a digital hand dynamometer while cognitive function was assessed via a consensus diagnosis. The relationship between cognitive impairment and HGS was investigated using a multivariable-adjusted logistic regression analysis.

**Results:**

The mean (SD) age of the study participants was 64.6 ± 11.5 years and 67.6% were females. The proportion of participants with cognitive dysfunction was 22.9%, while the mean (SD) HGS (in kg) was 18.16 (8.06). The mean (SD) HGS was lower among participants with cognitive dysfunction (13.44 ± 5.45) compared to those without cognitive impairment (19.58 ± 8.38; p-value <0.001). After adjustment for age, sex, clinical frailty, level of education, and other metabolic risk markers, high HGS showed a protective association with cognitive impairment, aORs (95%CI) 0.91 (0.87–0.95). This protective association [aORs (95% CI)] was consistent for individuals aged <65 years [0.87 (0.80–0.94] and ≥65 years [0.90 (0.85–0.96)], as well as males 0.88 (0.78–0.99) and females 0.91 (0.84–0.99).

**Conclusions:**

HGS was independently associated with cognitive impairment, buttressing the intricate link between physical and cognitive health in this unique West African population. Future work will explore the predictive ability of grip strength as an early indirect non-invasive biomarker of incident cognitive decline and the utility of targeted resistance training exercises in the primary prevention of neurocognitive disorders.

## Introduction

Globally, dementia is a major cause of death, disability, and dependency. The number of people living with dementia is likely to triple from the current 57.4 million to over 150 million by 2050 with the greatest increases in Africa and Asia – largely attributable to population growth and cardiometabolic disorders [1,2]. Alzheimer’s disease and related dementias once thought to be rare, are now a major public health priority with substantial adverse impact on individuals, families and caregivers, healthcare systems, and the community as a whole [1,2]. To reduce this burden, and for effective, personalized, timely targeted interventions, innovative, cost-effective, and culturally sensitive screening tools will be required for early identification of individuals with dementia or those at risk [3].

Frailty has emerged as an important construct in geriatric medicine in recent years. According to the Fried model, hand grip strength (HGS) is one of the five components of frailty. Hand-grip strength (HGS) is a known predictor of frailty and fitness, cardiometabolic, and cognitive health [3–8]. There is a plethora of evidence that suggests that measures of motor function and physical fitness such as muscle strength, gait speed, and other measures of physical performance may be useful biomarkers to predict the incidence of cognitive health or disease [9,10]. Changes in the performance of motor tasks and physical functioning have been associated with changes in cognition and are a harbinger of impending cognitive dysfunction [11,12].

The relationship between physical and cognitive health has been attributed to several pathobiological mechanisms and hypothetical pathways. One such hypothesis is a suggestion that both cognitive and motor function share similar neuro-cortical pathways such that, any disruption in the shared neuro-cortical axis may result in concomitant deficits in both cognitive and motor performance. In addition, deep white matter and subcortical hyperintensities, known neuropathological substrate of cognitive impairment, have been associated with greater cognitive and muscular mass loss as well as slowing of gait speed [13,14]. Furthermore, participation in tailored physical activity and resistance exercises not only improves physical fitness but it also appears to help maintain robust brain health functions by improving microcirculatory cerebral blood flow [15]. Lifelong adherence to minimum recommended physical activity has been shown to be associated with improved neuronal integrity in old age [15]. Lastly, it has similarly been opined that increased protein oxidation that occurs as one gets older leads to cellular dysfunction and a reduction in tissue function.

Despite these shared pathobiological pathways, the relationship between HGS and cognitive impairment yet varies according to genetic, geographic, ethno-racial, and socio-cultural affiliations [6,16,17]. Similar to other complex traits, genetic variants of small effect sizes influence HGS [17], heightening curiosity for variability across ancestry. In addition, HGS can be readily deployed as a low-cost screening tool in resource-limited settings where multi-modal mechanisms aid primordial, primary, and secondary prevention of dementia. [18,19] However, robust data supporting the relationship of HGS with cognitive health in such settings are lacking. A recent study noted that HGS was inversely associated with all-cause cardiovascular and non-cardiovascular mortality in high-income countries, but the link with non-cardiovascular disorders was inconsistent in middle-income and low-income countries (LMICs) [18,20]. An understanding of the relationship between HGS, frailty, and cognitive impairment among elderly West Africans, known for a rising incidence of neurocognitive and cardiometabolic disorders, may therefore help change this narrative [6,21–23]. We investigated the relationship between HGS, cardiometabolic, and cognitive impairment among older adults in an urban community in Ibadan, South West Nigeria.

## Methods

### Study population

TheVascular heAlth, fraiLty and cognItion in Ageing Nigerians sTudy (VALIANT) is a longitudinal community-based cohort study aimed at exploring the association between cardiovascular health, cognition, and frailty markers in Nigeria [24,25]. A multistage sampling method was employed to recruit 1021 study participants from an urban community in Yemetu, Ibadan North Local Government Area, Oyo State, South West Nigeria over 12 months. The first participant was recruited on 1st November 2021 while the last participant was recruited on 30th October 2022. Using the African Rigorous Innovative Stroke Epidemiological Surveillances (ARISES) database [26] as a sampling frame, two wards (Ward 3 & 4) were purposively selected. ARISES is an ongoing observational cohort study in selected wards in Ibadan North and Ibarapa Central local government areas in Oyo State, Nigeria. Wards 3 & 4 were purposively selected for the VALIANT study because they host more Indigenous urban dwellers than Ward 1, which is predominantly a government reservation area for offices and other establishments. Thereafter, 11 out of the 16 clusters from the two Wards were randomly selected and all households within the selected clusters were visited to recruit eligible participants. A total of thousand twenty-one (1021) participants were enrolled and taken through a battery of cardiovascular, cognitive, and frailty assessment tools. Ethical approval was obtained from the University of Ibadan/University College Hospital (UI/UCH) Health Research Ethics Committee (HREC) with IRB no: UI/EC/20/0508. Participation in this study was voluntary and without coercion or undue inducement after written informed consent was obtained.

### Sociodemographic data

Sociodemographic data were collected from all participants, including sex (men and women), age, level of education (up to primary [< 8 years of studies], secondary [between 8 and 12 years of studies], and beyond secondary [> 12 years of study]), marital status (married and others that includes single, widowed or separated) and, Yoruba ethnicity (yes or no).

### Cardiovascular health (CVH)/risk factors estimation

Components of the CVH metric include blood pressure, fasting glucose, total cholesterol, BMI, physical activity, diet, and smoking. Systolic blood pressure and diastolic blood pressure were measured by a mercury sphygmomanometer on the right arm with the subject in a sitting position after 10 min of rest. The average of two measurements 5 min apart was used in the statistical analyses. Venous blood samples were drawn for the measurement of glucose and lipid profiles after an overnight fast. Fasting lipid profile and fasting plasma glucose were assessed using standard laboratory techniques. BMI was calculated from the height and weight measurements of participants. Physical activity, smoking status, and dietary pattern were assessed using a standardized questionnaire [27,28].

### Assessment of cognition, grip strength, and frailty

Cognitive function was assessed using translated and validated neuro-psychometric instruments: Montreal Cognitive Assessment [29], and the Identification and Intervention for Dementia in Elderly Africans (IDEA) cognitive screen [30]. The IDEA and MoCA are tests of general cognitive functioning that have been well-validated in the African setting. Patients were initially screened using the IDEA (<9), MoCA (<19), scores, and functional impairment assessment using the IDEA-ADL (<11) and FAS scores (>9). They were then diagnosed as having cognitive impairment (MCI and dementia) via a consensus diagnosis of two neurologists. The clinical frailty scale was calculated using the Rockwood scale [31]. Data on HGS from 608 participants were obtained using a digital hand dynamometer. Grip strength was measured twice by trained research assistants for both hands with the use of Smedley’s digital hand dynamometer. If the participant had a stroke with hand/arm weakness, any surgery in the last 3 months, or arthritis or pain in the hand/wrist/arm, grip strength was not measured in that hand.

### Statistical analyses

Continuous variables were described as mean± standard deviation (SD) while categorical variables were reported as frequencies/percentages. Cognitive impairment was defined using the MoCA and IDEA scores. Patients were screened using the MoCA and IDEA scores and then diagnosed as having cognitive impairment (dementia and MCI) via a consensus diagnosis of at least two neurologists. To ensure robust characterization of the relationship between hand grip strength (HGS) and cognitive function, HGS was specified as a continuous variable, while adjusting for documented confounders like age, sex, level of education, cardiovascular, lifestyle as well as clinical frailty indices. Adjusted multivariate logistic regression analysis was performed to identify factors independently associated with cognitive impairment. The models were then stratified by age 65, to explore age-related differentials and by sex, to explore sex-related differentials in patterns of relationships. The strengths and directions of the relationships were reported as odds ratios (ORs) with a 95% confidence interval (CI). All statistical analyses were conducted using Stata SE version 16 with a significance level set at 0.05.

## Results

### Baseline characteristics

Of the 608 study subjects with HGS data, the mean (SD) age was 64.6 (11.5) years and 67.6% were females (**Table 1**). There was no statistically significant difference in the mean (SD) age (in years) or ethnicity of participants with hand grip data compared to those without hand grip data (Table S1 in S1 File). The proportion of participants with cognitive dysfunction was 22.9%, while the mean (SD) HGS (in kg) was 18.16 (8.06) (**Table 1**). The mean (SD) HGS (in kg) was lower among participants with cognitive dysfunction (13.44 ± 5.45) compared to those without cognitive impairment (19.58 ± 8.38; p-value <0.001) (Table S2 in S1 File). There was a significant bivariate relationship between hand grip strength as well as age, sex, level of education, marital status, living situation, and frailty (**Table 1).** Female participants had a lower mean (SD) HGS (in kg) (16.01 + 6.02) compared to male participants (22.63 ± 9.76; p-value <0.001). Similarly, individuals with no education had the lowest mean (SD) HGS (in kg) 14.54 ± 6.09 compared to those with primary education 18.47 ± 7.09, secondary education 20.92 ± 8.94, and tertiary education 23.17 ± 11.64 p-value <0.001.

**Table 1:** Sample characteristics of the VALIANT cohort (n = 608).

	Total	Handgrip Strength, kg	p-value
Age (years)	64.6 ± 11.5		
Age category			
< 65	53.3	20.06 ± 8.42	<0.001
≥ 65	46.7	16.06 ± 7.05	
Sex			
Male	32.4	22.63 ± 9.76	<0.001
Female	67.6	16.01 ± 6.02	
Highest Level of Education			
None	29.6	14.54 ± 6.09	<0.001
Primary	40.5	18.47 ± 7.09	
Secondary	24.4	20.92 ± 8.94	
Tertiary	5.4	23.17 ± 11.64	
Marital status			
Currently married	47.7	20.66 ± 8.29	<0.001
Widow/widower	37.5	14.83 ± 5.76	
Separated/Single	14.8	18.50 ± 9.23	
Religion			
Christianity	46.1	18.43 ± 7.77	0.425
Islam	53.9	17.90 ± 8.31	
Living situation			
Lives alone	21.0	16.47 ± 8.44	<0.001
Lives with spouse and children	30.9	21.39 ± 9.14	
Lives with spouse	12.2	19.66 ± 7.03	
Lives with extended family	9.8	14.83 ± 6.10	
Lives with children	26.0	16.45 ± 5.92	
Ethnicity			
Yoruba	97.7	18.13 ± 8.10	0.564
Non-Yoruba	2.3	19.38 ± 5.26	
Diabetes			
No	94.7	18.25 ± 8.05	0.245
Yes	5.3	16.55 ± 7.96	
Hypertension			
No	37.3	17.96 ± 9.39	0.637
Yes	62.7	18.28 ± 7.14	
Alcohol use			
Never	64.1	16.83 ± 6.71	<0.001
Ever/currently using	35.9	20.59 ± 9.58	
Dyslipidaemia			
No	64.8	18.52 ± 8.60	0.132
Yes	35.2	17.49 ± 6.90	
Smoke			
No	89.3	17.79 ± 8.02	0.001
Yes	10.7	21.21 ± 7.70	
Body Mass Index			
Underweight	16.7	19.15 ± 7.66	0.319
Normal	37.3	17.52 ± 8.04	
Overweight	23.5	18.77 ± 9.60	
Obese	22.5	18.05 ± 6.69	
Clinical Frailty			
Very fit	9.6	20.46 ± 7.69	<0.001
Well	35.8	20.31 ± 9.47	
Managing Well	41.1	17.59 ± 6.91	
Vulnerable	8.7	13.12 ± 5.74	
Mildly frail	2.9	13.04 ± 6.30	
Moderately frail	1.4	10.50 ± 4.70	
Severely frail	0.2	16.07 ± 0	
Very severely frail	0.2	5.00 ± 0	
Cognitive impairment			
Normal	77.1	19.58 ± 8.38	<0.001
Impaired	22.9	13.44 ± 5.45	

* P- value<0.05 considered significant.

There was no significant bivariate relationship between the presence of hypertension and HGS. Participants with dyslipidemia had a lower mean (SD) HGS of 17.49 ± 6.90 compared to those without dyslipidemia with an HGS of 18.52 ± 8.60; while participants with T2DM similarly had a lower mean (SD) HGS of 16.55 ± 7.96 compared to those without T2DM 18.25 ± 8.05, although not statistically significant. In the bivariate analysis, HGS was negatively related to alcohol or smoking status; individuals who smoked and consumed alcohol had higher HGS than those who did not. Clinical frailty showed a significant relationship with HGS as very fit individuals had higher mean (SD) HGS (20.46 ± 7.69 kg) compared to the other frailty categories who had significantly lower HGS **Table 1**.

### Unadjusted logistic regression

The linear regression is shown in **Table 2**. Overall, co-variates with a significant positive univariate relationship with cognitive impairment were older age 1.10 (1.07–1.12), female sex 2.46 (1.51–4.01), and higher grades of clinical frailty. Participants who were vulnerable, mildly frail, and moderately frail were more likely to have cognitive impairment. The respective ORs (95%CI) were determined to be 4.75 (1.85–12.21), 5.62 (1.63–19.34), and 16.87 (2.87–98.89). Educational attainment (tertiary, secondary, and primary), living situation (living with spouse and children) and higher BMI categories showed a protective association with cognitive impairment as shown in Table 2. Hand grip strength (HGS) showed a protective association with cognitive impairment with OR (95%CI) 0.87 (0.84–0.90) and this was consistent among participants <65 years 0.91 (0.86–0.96) and those ≥65 years 0.86 (0.82–0.91). As shown in supplementary Tables S3a and S3b in S1 File, the relationship between age, clinical frailty scale (CFS) score as well as HGS was similarly consistent between male and female participants.

**Table 2:** Univariable model of the correlates of cognitive impairment (N = 608).

	Overall		Age < 65		Age ≥ 65	
	OR	95% CI	OR	95% CI	OR	95% CI
Age (years)	1.10	1.07-1.12*	1.04	0.95-1.14	1.09	1.05-1.13*
Sex						
Male	Ref		Ref		Ref	
Female	2.46	1.51-4.01*	1.91	0.74-4.93	2.61	1.42-4.77*
Highest Level of Education						
None	Ref		Ref		Ref	
Primary	0.24	0.15-0.39*	0.71	0.25-2.02	0.26	0.15-0.48*
Secondary	0.10	0.05-0.21*	0.24	0.07-0.86*	0.34	0.12-0.94*
Tertiary	0.16	0.05-0.49*	0.27	0.03-2.45	0.28	0.07-1.11
Marital status						
Currently married	Ref		Ref		Ref	
Widow/widower	4.29	2.68-6.87*	2.04	0.74-5.60	2.50	1.37-4.56*
Separated/Single	2.77	1.46-5.24*	4.37	1.65-11.53	1.45	0.58-3.61
Religion						
Christianity	Ref		Ref		Ref	
Islam	1.41	0.94-2.11	1.84	0.79-4.28	1.15	0.69-1.91
Living situation						
Lives alone	Ref		Ref		Ref	
Lives with spouse and children	0.12	0.05-0.27*	0.23	0.06-0.79	0.22	0.06-0.70*
Lives with spouse	0.59	0.29-1.21	1.23	0.33-4.48	0.44	0.17-1.08
Lives with extended family	1.60	0.82-3.11	0.94	0.16-5.30	1.38	0.63-3.04
Lives with children	0.94	0.55-1.61	0.83	0.26-2.60	1.03	0.53-1.99
Ethnicity						
Yoruba	Ref		Ref		Ref	
Non-Yoruba	1.01	0.27-3.71	–		0.63	0.12-3.31
Diabetes						
No	Ref		Ref		Ref	
Yes	0.79	0.31-1.98	0.79	0.09-6.32	0.64	0.22-1.89
Hypertension						
No	Ref		Ref		Ref	
Yes	1.28	0.84-1.95	1.27	0.55-2.90	0.85	0.49-1.48
Alcohol use						
Never	Ref		Ref		Ref	
Ever/currently using	0.60	0.38-0.93*	1.14	0.49-2.67	0.48	0.27-0.85*
Dyslipidaemia						
No	Ref		Ref		Ref	
Yes	0.93	0.61-1.41	0.83	0.35-1.99	0.96	0.57-1.63
Smoke						
No	Ref		Ref		Ref	
Yes	0.37	0.16-0.83*	–	–	0.50	0.19-1.31
Body Mass Index						
Underweight	Ref		Ref		Ref	
Normal	1.09	0.61-1.91	2.03	0.54-7.55	0.86	0.43-1.73
Overweight	0.57	0.29-1.12	0.97	0.22-4.30	0.54	0.23-1.25
Obese	0.56	0.28-1.11	0.70	0.14-3.30	0.75	0.31-1.77
Clinical Frailty						
Very fit	Ref		Ref		Ref	
Well	0.74	0.31-1.77	0.60	0.19-1.86	0.45	0.08-2.31
Managing Well	1.89	0.84-4.26	1.00	0.32-3.11	0.69	0.14-3.25
Vulnerable	4.75	1.85-12.21*	–	–	1.39	0.27-7.00
Mildly frail	5.62	1.63-19.34*	2.73	0.23-31.55	1.60	0.23-10.80
Moderately frail	16.87	2.87-98.89*	–	–	4.00	0.44-35.78
Severely frail	–	–	–	–	–	–
Very severely frail	–	–	–	–	–	–
Grip	0.87	0.84-0.90*	0.91	0.86-0.96*	0.86	0.82-0.91*

* P- value<0.05 considered significant.

### Adjusted logistic regression

In the multivariate-adjusted logistic regression analysis, overall, significant independent determinants of cognitive impairment with respective aORs (95% CI) were older age 1.07 (1.03–1.10), attainment of primary level of education 0.34 (0.16–0.75) or secondary education 0.35 (0.14–0.86), and higher hand grip strength 0.91 (0.87–0.95) **(**Table 3**)**. For individuals <65 years of age and those aged≥65 years, HGS showed a consistent protective association with cognitive impairment with aORs (95% CI) 0.87 (0.80–0.94) and 0.90 (0.85–0.96) respectively **(**Table 3**)**. As shown in supplementary Tables S4a and S4b in S1 File, the independent protective relationship between HGS and cognitive dysfunction was similarly sustained between male 0.88 (0.78–0.99) and female 0.91 (0.84–0.99) participants.

**Table 3:** Multivariable Model of the Correlates of Cognitive Impairment (N = 608).

	Overall		Age < 65		Age ≥ 65	
	OR	95% CI	OR	95% CI	OR	95% CI
Age (years)	1.07	1.03-1.10*	0.98	0.89-1.08	1.07	1.02-1.13*
Sex						
Male	ref		Ref		Ref	
Female	0.86	0.38-1.92	0.33	0.08-1.37	1.03	0.36-2.93
Highest Level of Education						
None	Ref		Ref		Ref	
Primary	0.44	0.23-0.82*	1.15	0.30-4.28	0.34	0.16-0.75*
Secondary	0.35	0.14-0.86*	0.47	0.09-2.48	0.44	0.12-1.59
Tertiary	0.56	0.14-2.23	1.36	0.11-16.54	0.93	0.14-5.88
Marital status						
Currently married	Ref		Ref		Ref	
Widow/widower	0.84	0.31-3.25	1.24	0.17-8.95	0.44	0.12-1.60
Separated/Single	1.79	0.63-5.12	5.13	0.75-35.16	0.55	0.13-2.34
Living situation						
Lives alone	Ref		Ref		Ref	
Lives with spouse and children	0.36	0.10-1.24	1.08	0.10-10.84	0.29	0.05-1.54
Lives with spouse	1.24	0.38-4.08	10.49	0.95-115.19	0.59	0.12-2.75
Lives with extended family	1.65	0.72-3.76	3.04	0.34-27.12	1.81	0.69-4.74
Lives with children	1.23	0.62-2.43	3.79	0.69-20.88	1.33	0.60-2.94
Alcohol use						
Never	Ref		Ref		Ref	
Ever/currently using	1.43	0.74-2.77	2.10	0.72-6.10	1.06	0.44-2.58
Smoke						
No	Ref		Ref		Ref	
Yes	0.46	0.15-1.39	–		1.31	0.35-4.84
Clinical Frailty						
Very fit	Ref		Ref		Ref	
Well	0.33	0.11-0.92*	0.23	0.05-1.01	0.41	0.06-2.46
Managing Well	0.39	0.14-1.08	0.62	0.15-2.54	0.41	0.07-2.24
Vulnerable	0.38	0.11-1.31	–		0.41	0.06-2.57
Mildly frail	0.57	0.11-2.94	1.46	0.09-23.51	0.54	0.05-5.40
Moderately frail	0.67	0.08-5.36	–		0.63	0.05-8.08
Severely frail	–		–		–	
Very severely frail	–		–		–	
Grip	0.91	0.87-0.95*	0.87	0.80-0.94*	0.90	0.85-0.96*

* P- value<0.05 considered significant.

## Discussion

In the VALIANT study, we have built a population-based cohort of approximately 1000 community-dwelling older adults in Ibadan South-west Nigeria, and characterised the cohort fully in terms of cardiovascular, cognitive, and frailty status at baseline. While the index study examined the relationship between HGS and cognitive function among 608 participants, subjects with HGS did not differ by age or ethnicity compared to those without HGS. Overall, the mean HGS was lower among participants with cognitive impairment as compared to those without cognitive impairment. More specifically, a higher HGS was associated with lower odds of cognitive impairment having adjusted for age, sex, level of education, marital status, clinical frailty, hypertension, and alcohol use. While the protective association of high HGS was stronger among participants less than 65 years, it was yet consistent among those aged≥65 years. Similarly, there was no observed sex-differential in the protective association between HGS and cognitive dysfunction. Our study expands the frontiers of understanding espousing the link between frailty and cognitive decline among indigenous Africans in LMICs and suggests that a cheap and readily available measurement of handgrip strength may help to identify persons who are likely to have cognitive impairment. Given the rapidly changing population demographics and increase in clusters of vascular risk factors for dementia in many LMICs, as well as low levels of education, social isolation, and socioeconomic stress, our findings underline a need to prioritize, further investigate, and intervene in frailty as a potential risk factor for cognitive impairment in low-resource settings. Potential strategies may involve targeted improvements in physical as well as nutritional rehabilitation.

While yet inconclusive, the exact nature of the relationship between cognition and HGS can be described as bidirectional [32,33]. It has been shown that weak HGS may precede the onset of dementia and MCI [34–37] and vice versa [32,33,38]. Among 2160 non-institutionalized Mexican Americans aged ≥65 years and followed up for 7 years, low baseline handgrip strength significantly predicted a decline in cognitive performance [39]. In another prospective study, handgrip strength predicted an accelerated 1-year decline in cognitive function, in a cohort of 104 older adults, followed up for 11 months [40]. Among over 10,000 Korean adults, males in the highest quartile of the HGS were 71.9% less likely to experience cognitive impairment than those in the lowest quartile [37]. The odds of cognitive impairment for men in the third and second quartiles reduced by 62.6% and 60.4% respectively. The odds of cognitive impairment for women declined to 72.7%, 63.0%, and 41.8% for the fourth, third, and second quartiles, respectively, compared with the lowest quartile [37]. A recent longitudinal study of 3099 community‐dwelling older adults assessed physical performance measures such as short Physical Performance Battery (SPPB), 4 m gait speed, chair stands time, leg extension and flexion, handgrip strength, and 6-Minute Walking Test (6MWT) [9]. The authors showed slow walking speed as an independent predictor of poor cognitive status over a 4.4-year follow-up, while other items of SPPB were also significantly associated with CI [9]. These findings, shown in non-African populations, buttress our observations in the index study.

In a cross-sectional, community-based analysis of precisely 32,000 individuals aged 50 years or older, participants with MCI were more likely to have weak handgrip, having taken account for confounding variables [36]. On the flip side, greater degrees of cognitive loss or advanced dementia may also be associated with reduced grip strength [33,41]. Since motor skill learning and motor output are dependent on the activities of the frontal and parietal cortex, disruption of the interconnection between these brain regions in advanced dementia may be due to reduced motor output [33]. A recent longitudinal study of 708 adults assessed at six-time points over 20 years noted that grip strength performance was associated with a change in the 4 cognitive abilities of verbal ability, spatial ability, processing speed, and memory after age 65 years [34].

Since HGS is similarly a surrogate marker of cardiometabolic health18, patients with low HGS and cognitive impairment have been shown to have a higher risk for cardiometabolic diseases and low physical activity [42], which are on their turn risk factors for further cognitive decline, disability, and death [20,43,44]. In our study, while participants with dyslipidemia and diabetes mellitus (DM) had low mean HGS, there was no significant bivariate relationship between the presence of dyslipidemia, DM, or hypertension and HGS. This may be due to the low prevalence of cardiometabolic disturbance in this population-based, community-dwelling cohort. Persons with chronic metabolic conditions and multimorbidity are significantly less physically active and hence tend to be frail [45]. In our study, the association between handgrip strength and cognitive impairment remained significant even after controlling for frailty and chronic metabolic conditions such as hypertension, diabetes, obesity, and dyslipidemia, as well as behavioral risk markers like smoking, and alcohol use. We similarly controlled for other risk factors like social isolation, marital status, living situation, age, sex level of education, and physical inactivity indicating that the observed relationship is likely to be explained by other underlying biological mechanisms. A recent longitudinal study indeed observed that the link between HGS and cognition may be mediated by functional limitation [46].

While the exact pathobiological basis of the association between physical and cognitive health remains largely unknown, some authors have posited that frailty and cognitive impairment may share a common patho-mechanistic pathway. For example, human striated skeletal muscle, now widely regarded as an endocrine and immunogenic organ, is the target of numerous neuro-hormones [47]. This understanding provides a conceptual framework and a new paradigm for understanding how skeletal muscles interact with other organs such as adipose tissue, liver, pancreas, bones, and brain [48]. Myokines may likely contribute to the mediation of the health benefits of exercise [49]. Emerging evidence suggests a role of skeletal muscles in the secretion of certain cytokines, peptides, cytokines such as brain‐derived neurotrophic factor (BDNF), and several interleukins (IL‐6, IL‐8, and IL‐15). Findings from recent systematic reviews and meta-analysis suggest that sarcopenia and frailty may be associated with an inflammatory state [50–52], – noted to be linked with dementia [53,54]. A decline in muscle mass and strength may reduce the expression of BDNF, thought to play a role in brain health [48,55].

Deficiency of vitamin D deficiency has similarly been invoked as a potential biological factor in the causal link between movement, mood, and memory [56]. There are reports on associations with frailty, cardiovascular health, and cognitive performance [57,58]. Results from prospective cohort studies and randomized controlled trials of vitamin D supplementation are yet inconclusive. While some evidence suggests that low serum 25‐hydroxyvitamin D (25(OH)D) may impact cognitive health [59,60], other studies show no association [61]. In a cohort of 509 adults, patients with Alzheimer’s dementia and mixed dementia showed the lowest vitamin D levels, while MCI patients showed higher levels than the other groups60. Similarly, there are clinical and pre-clinical documented associations between low serum 25(OH)D concentrations, frailty, and handgrip strength [62,63]. This is mediated via the impact of low vitamin D on calcium homeostasis and musculoskeletal health. Prospective cohort as well as interventional studies are however yet needed to validate or refute this hypothesis, which may potentially uncover poor nutrition in the causal pathways between physical and cognitive health [64].

Physical and cognitive training may delay dementia in later life but the neural mechanisms underlying these therapeutic benefits require further study [65]. Future research efforts should investigate how increasing muscle fitness and reducing frailty may be a therapeutic target for improving cognitive and functional outcomes of those with MCI in LMICs. Among the elderly population, there is a handful of evidence from multiple randomized controlled trials that sustained moderate- or high-intensity resistance training significantly improves cognitive functioning, with domain-specific improvement noted in executive function [66–68]. Acute aerobic exercises have not yielded particularly significant results on cognitive flexibility [69]. It has however been postulated that the potentially helpful benefits of resistance training and exercise may be mediated through increases in grey matter volume and attenuation of aging‐related white matter abnormalities which are typically exercise-mediated [68,70]. Physical and cognitive exercises rely on discrete neuronal physiologic mechanisms for their therapeutic efficacy71, knowledge of which may form the basis for developing targeted, preventative strategies to reduce the burden of dementia in LMICs [71].

### Limitations and strength

Our study has several strengths. One of these is the large sample size, giving a robust characterization as a pioneering effort investigating the relationship between vascular health, and physical and cognitive health among indigenous Nigerian Africans. Our findings are potentially generalizable to not only West Africans but other regions in sub-Saharan Africa. Secondly, the diagnosis of cognitive impairment was done via a consensus diagnosis involving two neurologists, following screening with two cognitive batteries. Our study is however not without limitations. This was a cross‐sectional study and therefore causality cannot be strictly inferred. Although our data offer relevant hypotheses to address frailty as a risk factor for cognitive impairment among Indigenous Africans, follow-up cohort studies will be needed to better understand the relationship between HGS and cognitive impairment. Moreover, larger female participation in this study may have led to lower overall mean values of HGS in the population. We however note that the female sex is at a higher risk of MCI and dementia and this may have enabled better characterization of the observed relationship between HGS and cognitive function. While handgrip data were available for 608 participants from this cohort of about 1000 participants, our findings are generalizable to the subset without HGS data given the similar age bracket, ethnicity, and better male representation.

## Conclusion and future directions

In conclusion, our study shows that individuals with cognitive impairment have lower HGS. We also showed that HGS is independently associated with cognitive impairment, having adjusted for age, sex, clinical frailty, and other metabolic risk markers. The independent relationship between HGS and cognitive function buttresses the intricate link between physical and cognitive health in this unique West African population. Future efforts should therefore explore the predictive ability of grip strength as an early indirect, non-invasive biomarker of pre-clinical or incident cognitive decline, in resource-limited settings. Handgrip dynamometers are affordable, inexpensive, portable, non-invasive, fast, reliable, and do not require formal, extensive training for routine use. They can be readily deployed to provide useful baseline (and/or follow-up) data, while evaluating the potential utility of resistance training regimen in neuro-cognitive and physical health, especially among historically understudied populations.

## Supporting information

S1 File This file contains Tables S1–S4, presenting participant characteristics with and without hand grip data (Table S1), comparisons of hand grip strength by cognitive status (Table S2), and sex-stratified univariable (Tables S3a and S3b) and multivariable models (Tables S4a and S4b) of correlates of cognitive impairment. (DOCX)

## References

[1] Akinyemi RO, Yaria J, Ojagbemi A, Guerchet M, Okubadejo N, Njamnshi AK, et al. Dementia in Africa: current evidence, knowledge gaps, and future directions. Alzheimers Dement. 2022;18(4):790–809. doi: 10.1002/alz.12432 34569714 PMC8957626

[2] Akinyemi RO, Owolabi MO, Okubadejo N, Ogunniyi A, Kalaria RN, African Dementia Consortium. The African dementia consortium. Lancet Neurol. 2023;22(1):28–9. 10.1016/S1474-4422(22)00475-6 PMC10022738 36517165

[3] Heward J, Stone L, Paddick S-M, Mkenda S, Gray WK, Dotchin CL, et al. A longitudinal study of cognitive decline in rural Tanzania: rates and potentially modifiable risk factors. Int Psychogeriatr. 2018;30(9):1333–43. doi: 10.1017/S1041610217002861 29559014

[4] Cui M, Zhang S, Liu Y, Gang X, Wang G. Grip strength and the risk of cognitive decline and dementia: a systematic review and meta-analysis of longitudinal cohort studies. Front Aging Neurosci. 2021;13:625551. 33613270 10.3389/fnagi.2021.625551 PMC7890203

[5] Su H, Sun X, Li F, Guo Q. Association between handgrip strength and cognition in a Chinese population with Alzheimer’s disease and mild cognitive impairment. BMC Geriatr. 2021;21(1):459. doi: 10.1186/s12877-021-02383-8 34380435 PMC8356394

[6] Ong HL, Abdin E, Chua BY, Zhang Y, Seow E, Vaingankar JA, et al. Hand-grip strength among older adults in Singapore: a comparison with international norms and associative factors. BMC Geriatr. 2017;17(1):176. doi: 10.1186/s12877-017-0565-6 28778190 PMC5544979

[7] Hamasaki H. What can hand grip strength tell us about type 2 diabetes?: mortality, morbidities and risk of diabetes. Expert Rev Endocrinol Metab. 2021;16(5):237–50. doi: 10.1080/17446651.2021.1967743 34402694

[8] Pan P-J, Hsu N-W, Lee M-J, Lin Y-Y, Tsai C-C, Lin W-S. Physical fitness and its correlation with handgrip strength in active community-dwelling older adults. Sci Rep. 2022;12(1):17227. doi: 10.1038/s41598-022-21736-w 36241763 PMC9568649

[9] Veronese N, Stubbs B, Trevisan C, Bolzetta F, De Rui M, Solmi M, et al. What physical performance measures predict incident cognitive decline among intact older adults? A 4.4year follow up study. Exp Gerontol. 2016;81:110–8. doi: 10.1016/j.exger.2016.05.008 27235850

[10] Boyle PA, Buchman AS, Wilson RS, Leurgans SE, Bennett DA. Physical frailty is associated with incident mild cognitive impairment in community-based older persons. J Am Geriatr Soc. 2010;58(2):248–55. doi: 10.1111/j.1532-5415.2009.02671.x 20070417 PMC3150526

[11] Cohen JA, Verghese J, Zwerling JL. Cognition and gait in older people. Maturitas. 2016;93:73–7. doi: 10.1016/j.maturitas.2016.05.005 27240713

[12] Grande G, Triolo F, Nuara A, Welmer A-K, Fratiglioni L, Vetrano DL. Measuring gait speed to better identify prodromal dementia. Exp Gerontol. 2019;124:110625. doi: 10.1016/j.exger.2019.05.014 31173841

[13] Boyle PA, Yu L, Fleischman DA, Leurgans S, Yang J, Wilson RS. White matter hyperintensities, incident mild cognitive impairment, and cognitive decline in old age. Ann Clin Transl Neurol. 2016;3(10):791–800. 27752514 10.1002/acn3.343 PMC5048389

[14] Ghanavati T, Smitt MS, Lord SR, Sachdev P, Wen W, Kochan NA, et al. Deep white matter hyperintensities, microstructural integrity and dual task walking in older people. Brain Imaging Behav. 2018;12(5):1488–96. doi: 10.1007/s11682-017-9787-7 29297156

[15] Engeroff T, Vogt L, Fleckenstein J, Füzéki E, Matura S, Pilatus U, et al. Lifespan leisure physical activity profile, brain plasticity and cognitive function in old age. Aging Ment Health. 2019;23(7):811–8. doi: 10.1080/13607863.2017.1421615 29293024

[16] Dodds RM, Syddall HE, Cooper R, Kuh D, Cooper C, Sayer AA. Global variation in grip strength: a systematic review and meta-analysis of normative data. Age Ageing. 2016;45(2):209–16. doi: 10.1093/ageing/afv192 26790455 PMC4776623

[17] Chan JPL, Thalamuthu A, Oldmeadow C, Armstrong NJ, Holliday EG, McEvoy M, et al. Genetics of hand grip strength in mid to late life. Age (Dordr). 2015;37(1):9745. doi: 10.1007/s11357-015-9745-5 25637336 PMC4312310

[18] Leong DP, Teo KK, Rangarajan S, Lopez-Jaramillo P, Avezum A Jr, Orlandini A, et al. Prognostic value of grip strength: findings from the Prospective Urban Rural Epidemiology (PURE) study. Lancet. 2015;386(9990):266–73. doi: 10.1016/S0140-6736(14)62000-6 25982160

[19] Manjavong M, So-Ngern A, Limpawattana P, Manomaiwong N, Kamsuanjig T, Khammak C, et al. The prevalence of low handgrip strength and its predictors among outpatient older adults in a tertiary care setting: a cross-sectional study. Geriatrics (Basel). 2022;7(4):74. doi: 10.3390/geriatrics7040074 35893321 PMC9326717

[20] Ruiz JR, Sui X, Lobelo F, Morrow JR, Jackson AW, Sjöström M. Association between muscular strength and mortality in men: prospective cohort study. BMJ. 2008;337(7661):a439. 10.1136/bmj.a439 PMC2453303 18595904

[21] Bindawas SM, Vennu V, Al-Orf SM, Alshammari SA, Al-Amoud MM, Calder PC, et al. Normative data for handgrip strength in saudi older adults visiting primary health care centers. Medicina (Kaunas). 2019;55(6):251. doi: 10.3390/medicina55060251 31174395 PMC6631678

[22] Akinyemi RO, Allan L, Owolabi MO, Akinyemi JO, Ogbole G, Ajani A, et al. Profile and determinants of vascular cognitive impairment in African stroke survivors: the CogFAST Nigeria Study. J Neurol Sci. 2014;346(1–2):241–9. doi: 10.1016/j.jns.2014.08.042 25238666

[23] Akinyemi RO, Ovbiagele B, Adeniji OA, Sarfo FS, Abd-Allah F, Adoukonou T, et al. Stroke in Africa: profile, progress, prospects and priorities. Nat Rev Neurol. 2021;17(10):634–56. doi: 10.1038/s41582-021-00542-4 34526674 PMC8441961

[24] Akinyemi RO, Olalusi OV, Ogunde GO, Akinyemi TO, Yaria JO, Oguntiloye O, et al. Association between cardiovascular and cognitive health among older Indigenous Africans: data from an urban Nigerian settlement participating in the VALIANT study. Alzheimers Dement. 2025;21(9):e70669. doi: 10.1002/alz.70669 40968689 PMC12446708

[25] Akinyemi RO, Olalusi OV, Ogunde GO, Akinyemi TO, Yaria JO, Oguntiloye O, et al. Correlates of hand grip strength in a cohort of older Nigerian Africans: findings from the population-based VALIANT project. J Frailty Aging. 2025;14(5):100068. doi: 10.1016/j.tjfa.2025.100068 40906562 PMC12444469

[26] Popoola O, Ovbiagele B, Arulogun O, Akinyemi J, Akinyemi R, Uvere E, et al. African rigorous innovative stroke epidemiological surveillance: protocol for a community-based mobile-health study. Neuroepidemiology. 2022;56(1):17–24. doi: 10.1159/000518885 34903691 PMC9840813

[27] Keating XD, Zhou K, Liu X, Hodges M, Liu J, Guan J, et al. Reliability and Concurrent Validity of Global Physical Activity Questionnaire (GPAQ): a systematic review. Int J Environ Res Public Health. 2019;16(21):4128. doi: 10.3390/ijerph16214128 31717742 PMC6862218

[28] Akpalu A, Sarfo FS, Ovbiagele B, Akinyemi R, Gebregziabher M, Obiako R, et al. Phenotyping Stroke in Sub-Saharan Africa: Stroke Investigative Research and Education Network (SIREN) Phenomics Protocol. Neuroepidemiology. 2015;45(2):73–82. doi: 10.1159/000437372 26304844 PMC4604029

[29] Daniel B, Agenagnew L, Workicho A, Abera M. Psychometric Properties of the Montreal Cognitive Assessment (MoCA) to Detect major neurocognitive disorder among older people in Ethiopia: a validation study. Neuropsychiatr Dis Treat. 2022;18:1789–98. doi: 10.2147/NDT.S377430 36035074 PMC9416441

[30] Paddick S-M, Gray WK, Ogunjimi L, Lwezuala B, Olakehinde O, Kisoli A, et al. Validation of the Identification and Intervention for Dementia in Elderly Africans (IDEA) cognitive screen in Nigeria and Tanzania. BMC Geriatr. 2015;15:53. doi: 10.1186/s12877-015-0040-1 25908439 PMC4455989

[31] Moorhouse P, Rockwood K. Frailty and its quantitative clinical evaluation. J R Coll Physicians Edinb. 2012;42(4):333–40. doi: 10.4997/JRCPE.2012.412 23240122

[32] Cui M, Zhang S, Liu Y, Gang X, Wang G. Grip strength and the risk of cognitive decline and Dementia: a systematic review and meta-analysis of longitudinal cohort studies. Front Aging Neurosci. 2021;13:625551. doi: 10.3389/fnagi.2021.625551 33613270 PMC7890203

[33] Taekema DG, Ling CHY, Kurrle SE, Cameron ID, Meskers CGM, Blauw GJ, et al. Temporal relationship between handgrip strength and cognitive performance in oldest old people. Age Ageing. 2012;41(4):506–12. doi: 10.1093/ageing/afs013 22374646

[34] Sternäng O, Reynolds CA, Finkel D, Ernsth-Bravell M, Pedersen NL, Dahl Aslan AK. Grip strength and cognitive abilities: associations in old age. J Gerontol B Psychol Sci Soc Sci. 2016;71(5):841–8. 25787083 10.1093/geronb/gbv017 PMC6433432

[35] Zammit AR, Robitaille A, Piccinin AM, Muniz-Terrera G, Hofer SM. Associations between aging-related changes in grip strength and cognitive function in older adults: a systematic review. J Gerontol A Biol Sci Med Sci. 2019;74(4):519–27. doi: 10.1093/gerona/gly046 29528368 PMC6417444

[36] Vancampfort D, Stubbs B, Firth J, Smith L, Swinnen N, Koyanagi A. Associations between handgrip strength and mild cognitive impairment in middle-aged and older adults in six low- and middle-income countries. Int J Geriatr Psychiatry. 2019;34(4):609–16. doi: 10.1002/gps.5061 30672025

[37] Kim K, Kim H. Handgrip strength and cognitive function among elderly Koreans: insights from the Korean longitudinal study of ageing. Int J Environ Res Public Health. 2022;19(9):5262. doi: 10.3390/ijerph190925262 35564655 PMC9104585

[38] Firth J, Stubbs B, Vancampfort D, Firth JA, Large M, Rosenbaum S. Grip strength is associated with cognitive performance in schizophrenia and the general population: a UK Biobank study of 476559 participants. Schizophr Bull. 2018;44(4):728–36. 29684174 10.1093/schbul/sby034 PMC6007683

[39] Alfaro-Acha A, Al Snih S, Raji MA, Kuo Y-F, Markides KS, Ottenbacher KJ. Handgrip strength and cognitive decline in older Mexican Americans. J Gerontol A Biol Sci Med Sci. 2006;61(8):859–65. doi: 10.1093/gerona/61.8.859 16912105 PMC1635471

[40] Viscogliosi G, Di Bernardo MG, Ettorre E, Chiriac IM. Handgrip strength predicts longitudinal changes in clock drawing test performance. an observational study in a sample of older non-demented adults. J Nutr Health Aging. 2017;21(5):593–6. doi: 10.1007/s12603-016-0816-9 28448092 PMC12878403

[41] Taekema DG, Gussekloo J, Maier AB, Westendorp RGJ, de Craen AJM. Handgrip strength as a predictor of functional, psychological and social health. A prospective population-based study among the oldest old. Age Ageing. 2010;39(3):331–7. doi: 10.1093/ageing/afq022 20219767

[42] Koyanagi A, Stubbs B, Vancampfort D. Correlates of low physical activity across 46 low- and middle-income countries: a cross-sectional analysis of community-based data. Prev Med. 2018;106:107–13. doi: 10.1016/j.ypmed.2017.10.023 29066372

[43] Koyanagi A, Lara E, Stubbs B, Carvalho AF, Oh H, Stickley A, et al. Chronic physical conditions, multimorbidity, and mild cognitive impairment in low- and middle-income countries. J Am Geriatr Soc. 2018;66(4):721–7. doi: 10.1111/jgs.15288 29427504 PMC5906176

[44] Veronese N, Koyanagi A, Dominguez LJ, Maggi S, Soysal P, Bolzetta F, et al. Multimorbidity increases the risk of dementia: a 15 year follow-up of the SHARE study. Age Ageing. 2023;52(4):afad052. doi: 10.1093/ageing/afad052 37078753 PMC10116948

[45] Vancampfort D, Koyanagi A, Ward PB, Rosenbaum S, Schuch FB, Mugisha J, et al. Chronic physical conditions, multimorbidity and physical activity across 46 low- and middle-income countries. Int J Behav Nutr Phys Act. 2017;14(1):6. doi: 10.1186/s12966-017-0463-5 28100238 PMC5241915

[46] Chai S, Zhao D, Gao T, Wang X, Wang X, Luo J. The relationship between handgrip strength and cognitive function among older adults in China: Functional limitation plays a mediating role. J Affect Disord. 2024;347:144–9. 37992778 10.1016/j.jad.2023.11.056

[47] Nielsen S, Pedersen BK. Skeletal muscle as an immunogenic organ. Curr Opin Pharmacol. 2008;8(3):346–51. doi: 10.1016/j.coph.2008.02.005 18417420

[48] Pedersen BK, Febbraio MA. Muscles, exercise and obesity: skeletal muscle as a secretory organ. Nat Rev Endocrinol. 2012;8(8):457–65. doi: 10.1038/nrendo.2012.49 22473333

[49] Pedersen BK. Muscle as a secretory organ. Compr Physiol. 2013;3(3):1337–62. doi: 10.1002/cphy.c120033 23897689

[50] Shokri-Mashhadi N, Moradi S, Heidari Z, Saadat S. Association of circulating C-reactive protein and high-sensitivity C-reactive protein with components of sarcopenia: a systematic review and meta-analysis of observational studies. Exp Gerontol. 2021;150:111330. doi: 10.1016/j.exger.2021.111330 33848566

[51] Bano G, Trevisan C, Carraro S, Solmi M, Luchini C, Stubbs B, et al. Inflammation and sarcopenia: a systematic review and meta-analysis. Maturitas. 2017;96:10–5. doi: 10.1016/j.maturitas.2016.11.006 28041587

[52] Soysal P, Stubbs B, Lucato P, Luchini C, Solmi M, Peluso R, et al. Inflammation and frailty in the elderly: a systematic review and meta-analysis. Ageing Res Rev. 2016;31:1–8. doi: 10.1016/j.arr.2016.08.006 27592340

[53] Khatri A, Prakash O, Agarwal R, Kushwaha S. Systemic inflammatory markers and their association with Alzheimer’s disease: a cross-sectional analysis. Indian J Psychiatry. 2024;66(3):287–92. doi: 10.4103/indianjpsychiatry.indianjpsychiatry_975_23 39100114 PMC11293294

[54] Li C, Stebbins RC, Noppert GA, Carney CX, Liu C, Sapp ARM, et al. Peripheral immune function and Alzheimer’s disease: a living systematic review and critical appraisal. Mol Psychiatry. 2024;29(6):1895–905. doi: 10.1038/s41380-023-02355-x 38102484 PMC11483233

[55] Miranda M, Morici JF, Zanoni MB, Bekinschtein P. Brain-derived neurotrophic factor: a key molecule for memory in the healthy and the pathological brain. Front Cell Neurosci. 2019;13:363. doi: 10.3389/fncel.2019.00363 31440144 PMC6692714

[56] Houston DK. Vitamin D and age-related health outcomes: movement, mood, and memory. Curr Nutr Rep. 2015;4(2):185–200. 26779391 10.1007/s13668-015-0124-8 PMC4712711

[57] Toffanello ED, Coin A, Perissinotto E, Zambon S, Sarti S, Veronese N, et al. Vitamin D deficiency predicts cognitive decline in older men and women: The Pro.V.A. Study. Neurology. 2014;83(24):2292–8. 25378671 10.1212/WNL.0000000000001080

[58] Barnard K, Colón-Emeric C. Extraskeletal effects of vitamin D in older adults: cardiovascular disease, mortality, mood, and cognition. Am J Geriatr Pharmacother. 2010;8(1):4–33. doi: 10.1016/j.amjopharm.2010.02.004 20226390

[59] Aspell N, Lawlor B, O’Sullivan M. Is there a role for vitamin D in supporting cognitive function as we age? Proc Nutr Soc. 2018;77(2):124–34. doi: 10.1017/S0029665117004153 29233204

[60] Arosio B, Rossi PD, Ferri E, Cesari M, Vitale G. Characterization of Vitamin D status in older persons with cognitive impairment. Nutrients. 2022;14(6):1142. doi: 10.3390/nu14061142 35334800 PMC8949190

[61] Duchaine CS, Talbot D, Nafti M, Giguère Y, Dodin S, Tourigny A, et al. Vitamin D status, cognitive decline and incident dementia: the Canadian Study of Health and Aging. Can J Public Health. 2020;111(3):312–21. doi: 10.17269/s41997-019-00290-5 32016921 PMC7351937

[62] Haslam A, Johnson MA, Hausman DB, Cress ME, Houston DK, Davey A, et al. Vitamin D status is associated with grip strength in centenarians. J Nutr Gerontol Geriatr. 2014;33(1):35–46. doi: 10.1080/21551197.2013.867825 24597995

[63] Seldeen KL, Berman RN, Pang M, Lasky G, Weiss C, MacDonald BA. Vitamin D Insufficiency reduces grip strength, grip endurance and increases frailty in aged C57Bl/6J mice. Nutrients. 2020;12(10):3005. doi: 10.3390/nu12103005 33007912 PMC7599884

[64] Gojanovic M, Holloway-Kew KL, Hyde NK, Mohebbi M, Shivappa N, Hebert JR, et al. The dietary inflammatory index is associated with low muscle mass and low muscle function in older Australians. Nutrients. 2021;13(4):1166. doi: 10.3390/nu13041166 33916033 PMC8065722

[65] Gates NJ, Valenzuela M, Sachdev PS, Singh NA, Baune BT, Brodaty H, et al. Study of Mental Activity and Regular Training (SMART) in at risk individuals: a randomised double blind, sham controlled, longitudinal trial. BMC Geriatr. 2011;11:19. doi: 10.1186/1471-2318-11-19 21510896 PMC3110111

[66] Cassilhas RC, Viana VAR, Grassmann V, Santos RT, Santos RF, Tufik S, et al. The impact of resistance exercise on the cognitive function of the elderly. Med Sci Sports Exerc. 2007;39(8):1401–7. doi: 10.1249/mss.0b013e318060111f 17762374

[67] Liu-Ambrose T, Nagamatsu LS, Graf P, Beattie BL, Ashe MC, Handy TC. Resistance training and executive functions: a 12-month randomized controlled trial. Arch Intern Med. 2010;170(2):170–8. 20101012 10.1001/archinternmed.2009.494 PMC3448565

[68] Broadhouse KM, Singh MF, Suo C, Gates N, Wen W, Brodaty H, et al. Hippocampal plasticity underpins long-term cognitive gains from resistance exercise in MCI. Neuroimage Clin. 2020;25:102182. doi: 10.1016/j.nicl.2020.102182 31978826 PMC6974789

[69] Netz Y, Tomer R, Axelrad S, Argov E, Inbar O. The effect of a single aerobic training session on cognitive flexibility in late middle-aged adults. Int J Sports Med. 2007;28(1):82–7. doi: 10.1055/s-2006-924027 17213965

[70] Suo C, Singh MF, Gates N, Wen W, Sachdev P, Brodaty H, et al. Therapeutically relevant structural and functional mechanisms triggered by physical and cognitive exercise. Mol Psychiatry. 2016;21(11):1633–42. doi: 10.1038/mp.2016.19 27001615 PMC5078857

[71] Mavros Y, Gates N, Wilson GC, Jain N, Meiklejohn J, Brodaty H, et al. Mediation of cognitive function improvements by strength gains after resistance training in older adults with mild cognitive impairment: outcomes of the study of mental and resistance training. J Am Geriatr Soc. 2017;65(3):550–9. doi: 10.1111/jgs.14542 28304092

