# Supplementary material for: Hand grip strength and cognitive dysfunction amongst older Africans in Nigeria
Source: PLoS One. 2026 Feb 27;21(2):e0342598. doi: 10.1371/journal.pone.0342598 (PMC12948096; doi:10.1371/journal.pone.0342598)
Supplement: S1 File — (DOCX) [file pone.0342598.s001.docx]

**SUPPLEMENTARY FILE**

**S1 Table: Comparison of Characteristics of Participants with and without Hand Grip Data**

|  | Has Grip Data | |  |
| --- | --- | --- | --- |
|  | No (n=388; 39.0%) | Yes (n=608; 61.0%) | p-value |
| Age (Mean$\pm$SD) | 65.9$\pm$9.7 | 64.6$\pm$11.5 | 0.077 |
| Years of education | 5.1$\pm$4.8 | 6.1$\pm$5.2 | 0.002 |
| Gender |  |  |  |
| Male | 74(19.1) | 195(32.2) | <0.001 |
| Female | 314(80.9) | 410(67.8) |  |
| Religion |  |  |  |
| Christianity | 186(48.2) | 279(46.1) | 0.524 |
| Islam | 200(51.8) | 326(53.9) |  |
| Ethnicity |  |  |  |
| Yoruba | 372(95.9) | 594(97.7) | 0.101 |
| Non-Yoruba | 16(4.1) | 14(2.3) |  |
| Marital status |  |  |  |
| Currently married | 150(38.7) | 290(47.7) | <0.001 |
| Widow/widower | 198(51.0) | 228(37.5) |  |
| Others | 40(10.3) | 90(14.8) |  |
| Hypertensive (yes) | 218(56.2) | 381(62.7) | 0.042 |
| BMI | 24.06$\pm$6.17 | 26.49$\pm$7.26 | <0.001 |
| CFS | 2.36$\pm$1.03 | 2.65$\pm$1.01 | <0.001 |

**S2 Table: Comparison of Hand Grip Strength (HGS) Between Cognitively Impaired and Normal Participants**

|  | Mean$\pm$SD | 95% CI | t-value | p-value |
| --- | --- | --- | --- | --- |
| Normal | 19.58$\pm$8.38 | 18.79-20.38 | 7.80 | <0.001 |
| Impaired | 13.44$\pm$5.45 | 12.49-14.40 |  |  |
| Total | 18.18$\pm$8.22 | 17.49-18.86 |  |  |

**S3a Table: Univariable Model of the Correlates of Cognitive Impairment (Male Participants)**

| **Male** | **Overall** | | **Age <65** | | **Age** $\boldsymbol{\geq}$**65** | |
| --- | --- | --- | --- | --- | --- | --- |
|  | **OR** | **95% CI** | **OR** | **95% CI** | **OR** | **95% CI** |
| ***Age (years)*** | 1.09 | 1.04-1.14* | 0.78 | 0.56-1.08 | 1.13 | 1.04-1.24* |
| ***Highest level of education*** |  |  |  |  |  |  |
| None | Ref |  |  |  |  |  |
| Primary | 0.33 | 0.07-1.47 | 0.16 | 0.01-2.41 | 0.47 | 0.07-2.85 |
| Secondary | 0.13 | 0.02-0.74* | 0.05 | 0.01-1.10 | 0.42 | 0.04-3.79 |
| Tertiary | 0.30 | 0.04-2.11 | - |  | 0.75 | 0.08-6.95 |
| ***Marital status*** |  |  |  |  |  |  |
| Currently married | Ref |  |  |  |  |  |
| Widow/widower | 3.68 | 0.98-13.73 | - |  | 2.06 | 0.52-8.15 |
| Separated/single | 3.81 | 1.19-12.19* | - |  | 1.11 | 0.20-6.09 |
| ***Religion*** |  |  |  |  |  |  |
| Christianity | Ref |  |  |  |  |  |
| Islam | 0.48 | 0.17-1.31 | 2.04 | 0.20-20.34 | 0.30 | 0.08-1.09 |
| ***Living situation*** |  |  |  |  |  |  |
| Lives alone | Ref |  |  |  |  |  |
| Spouse and children | 0.06 | 0.01-0.53* | - |  | 0.26 | 0.02-2.74 |
| Spouse | 1.07 | 0.29-3.87 | - |  | 1.75 | 0.36-8.45 |
| Extended family | 3.60 | 0.67-19.16 | 2.83 | 0.19-41.99 | 4.22 | 0.48-36.76 |
| Children | 1.09 | 0.19-6.19 | - |  | 1.58 | 0.22-11.36 |
| ***Ethnicity*** |  |  |  |  |  |  |
| Yoruba | Ref |  |  |  |  |  |
| Non-Yoruba | - |  | - |  | - |  |
| ***Diabetes*** |  |  |  |  |  |  |
| No | Ref |  |  |  |  |  |
| Yes | 1.32 | 0.15-11.30 | - |  | 1.91 | 0.18-19.99 |
| ***Hypertension*** |  |  |  |  |  |  |
| No | Ref |  |  |  |  |  |
| Yes | 0.69 | 0.25-1.87 | - |  | 0.75 | 0.22-2.54 |
| ***Alcohol use*** |  |  |  |  |  |  |
| Never | Ref |  |  |  |  |  |
| Ever/currently using | 2.25 | 0.62-8.15 | 1.18 | 0.11-11.83 | 3.50 | 0.72-16.98 |
| ***Dyslipidaemia*** |  |  |  |  |  |  |
| No | Ref |  |  |  |  |  |
| Yes | 0.58 | 0.15-2.10 | - |  | 0.78 | 0.19-3.12 |
| ***Smoke*** |  |  |  |  |  |  |
| No | Ref |  |  |  |  |  |
| Yes | 0.96 | 0.32-2.89 | 0.64 | 0.06-6.45 | 1.38 | 0.37-5.07 |
| ***Body Mass Index*** |  |  |  |  |  |  |
| Underweight | Ref |  |  |  |  |  |
| Normal | 0.44 | 0.13-1.41 | 1.00 | 0.08-11.66 | 0.25 | 0.06-1.03 |
| Overweight | 0.24 | 0.04-1.27 | - |  | 0.23 | 0.03-1.44 |
| Obese | - |  | - |  | - |  |
| ***CFS*** |  |  |  |  |  |  |
| Well | Ref |  | Ref |  | Ref |  |
| Managing well | 1.81 | 0.46-7.07 | 1.40 | 0.12-16.30 | 1.12 | 0.18-6.75 |
| Vulnerable | 3.85 | 0.62-23.77 | - |  | 2.57 | 0.30-21.97 |
| Mildly frail | 9.62 | 0.71-129.89 | - |  | 4.50 | 0.27-74.74 |
| Moderately frail | 57.75 | 4.85-687.08* | - |  | 27.00 | 1.82-399.23* |
| Severely frail | - |  |  |  | - |  |
| ***HGS*** | 0.88 | 0.82-0.94* | 0.88 | 0.78-1.00 | 0.88 | 0.80-0.96* |

CFS, clinical frailty scale score; HGS, hand grip strength

**S3b Table: Univariable Model of the Correlates of Cognitive Impairment (Female Participants)**

| **Female** | **Overall** | | **Age <65** | | **Age** $\boldsymbol{\geq}$**65** | |
| --- | --- | --- | --- | --- | --- | --- |
|  | **OR** | **95% CI** | **OR** | **95% CI** | **OR** | **95% CI** |
| ***Age (years)*** | 1.10 | 1.07-1.13* | 1.05 | 0.90-1.22 | 1.08 | 1.03-1.12* |
| ***Highest level of education*** |  |  |  |  |  |  |
| None | Ref |  | Ref |  | Ref |  |
| Primary | 0.17 | 0.08-0.35* | 0.29 | 0.06-1.37 | 0.27 | 0.12-0.63* |
| Secondary | 0.06 | 0.01-0.26* | 0.13 | 0.01-1.24 | 0.25 | 0.03-2.15 |
| Tertiary | 0.26 | 0.03-2.12 | 1.28 | 0.12-13.29 | - |  |
| ***Marital status*** |  |  |  |  |  |  |
| Currently married | Ref |  | Ref |  | Ref |  |
| Widow/widower | 5.19 | 2.46-10.94* | 1.44 | 0.31-6.68 | 2.96 | 1.08-8.15* |
| Separated/single | 1.66 | 0.53-5.21 | 1.96 | 0.34-11.26 | 0.94 | 0.20-4.42 |
| ***Religion*** |  |  |  |  |  |  |
| Christianity | Ref |  | Ref |  | Ref |  |
| Islam | 1.43 | 0.83-2.46 | 2.29 | 0.55-9.44 | 1.05 | 0.56-1.99 |
| ***Living situation*** |  |  |  |  |  |  |
| Lives alone | Ref |  | Ref |  | Ref |  |
| Spouse and children | 0.13 | 0.04-0.42* | 0.23 | 0.03-1.50 | 0.46 | 0.08-2.39 |
| Spouse | 0.31 | 0.09-0.98* | 0.78 | 0.12-5.14 | 0.26 | 0.05-1.28 |
| Extended family | 1.06 | 0.48-2.33 | - |  | 0.99 | 0.41-2.39 |
| Children | 0.43 | 0.21-0.85* | 0.26 | 0.04-1.66 | 0.54 | 0.25-1.19 |
| ***Ethnicity*** |  |  |  |  |  |  |
| Yoruba | Ref |  | Ref |  | Ref |  |
| Non-Yoruba | 0.76 | 0.09-6.35 | - |  | 0.65 | 0.07-5.95 |
| ***Diabetes*** |  |  |  |  |  |  |
| No | Ref |  | Ref |  | Ref |  |
| Yes | 0.80 | 0.23-2.78 | - |  | 0.70 | 0.18-2.61 |
| ***Hypertension*** |  |  |  |  |  |  |
| No | Ref |  | Ref |  | Ref |  |
| Yes | 1.82 | 0.98-3.38 | 2.24 | 0.45-11.09 | 1.46 | 0.71-2.99 |
| ***Alcohol use*** |  |  |  |  |  |  |
| Never | Ref |  | Ref |  | Ref |  |
| Ever/currently using | 0.61 | 0.29-1.31 | 2.20 | 0.50-9.59 | 0.48 | 0.18-1.23 |
| ***Dyslipidaemia*** |  |  |  |  |  |  |
| No | Ref |  | Ref |  | Ref |  |
| Yes | 0.97 | 0.56-1.68 | 0.41 | 0.08-2.06 | 1.17 | 0.62-2.21 |
| ***Smoke*** |  |  |  |  |  |  |
| No | Ref |  | Ref |  | Ref |  |
| Yes | 0.54 | 0.06-4.29 | - |  | 1.33 | 0.11-15.05 |
| ***Body Mass Index*** |  |  |  |  |  |  |
| Underweight | Ref |  | Ref |  | Ref |  |
| Normal | 1.31 | 0.62-2.80 | - |  | 0.93 | 0.39-2.21 |
| Overweight | 0.32 | 0.11-0.87* | - |  | 0.38 | 0.12-1.13 |
| Obese | 0.20 | 0.07-0.58* | - |  | 0.27 | 0.09-0.84* |
| ***CFS*** |  |  |  |  |  |  |
| Very fit | 2.42 | 0.64-9.14 | 3.03 | 0.40-22.61 | 4.50 | 0.56-35.82 |
| Well | Ref |  | Ref |  | Ref |  |
| Managing well | 3.81 | 1.53-9.52* | 2.48 | 0.44-13.98 | 2.28 | 0.72-7.18 |
| Vulnerable | 10.73 | 3.68-31.30* | - |  | 4.38 | 1.24-15.48* |
| Mildly frail | 5.45 | 1.18-25.16* | - |  | 4.05 | 0.68-23.90 |
| Moderately frail | 54.49 | 4.90-605.45* | - |  | 20.25 | 1.67-245.44* |
| Severely frail | - | - | - |  | - |  |
| ***HGS*** | 0.86 | 0.82-0.91* | 0.94 | 0.84-1.04 | 0.85 | 0.79-0.92* |

CFS, clinical frailty scale score; HGS, hand grip strength

**S4a Table: Multivariable Model of the Correlates of Cognitive Impairment (Male Participants)**

| **Male** | **Overall** | | **Age <65** | | **Age** $\boldsymbol{\geq}$**65** | |
| --- | --- | --- | --- | --- | --- | --- |
|  | **OR** | **95% CI** | **OR** | **95% CI** | **OR** | **95% CI** |
| ***Age (years)*** | 1.07 | 0.98-1.16 | - |  | 1.16 | 0.99-1.36 |
| ***Highest level of education*** |  |  |  |  |  |  |
| None | Ref |  | Ref |  | Ref |  |
| Primary | 0.04 | 0.01-0.66* | - |  | 0.05 | 0.01-2.49 |
| Secondary | 0.11 | 0.01-1.94 | - |  | 0.07 | 0.01-4.58 |
| Tertiary | 0.17 | 0.01-4.45 | - |  | 0.27 | 0.01-22.44 |
| ***Marital status*** |  |  |  |  |  |  |
| Currently married | Ref |  | Ref |  | Ref |  |
| Widow/widower | 0.47 | 0.01-12.21 | - |  | 0.48 | 0.01-28.85 |
| Separated/single | 3.78 | 0.16-87.72 | - |  | 2.70 | 0.03-192.36 |
| ***Living situation*** |  |  |  |  |  |  |
| Lives alone | Ref |  | Ref |  | Ref |  |
| Spouse and children | - |  | - |  | - |  |
| Spouse | 2.90 | 0.11-73.45 | - |  | 4.94 | 0.04-495.40 |
| Extended family | 24.00 | 1.85-311.29* | - |  | 64.74 | 1.19-3521.08* |
| Children | 0.93 | 0.03-28.09 | - |  | 0.67 | 0.01-46.25 |
| ***CFS*** |  |  |  |  |  |  |
| Well | Ref |  |  |  | Ref |  |
| Managing well | 0.96 | 0.12-7.60 | - |  | 0.37 | 0.02-6.49 |
| Vulnerable | 1.17 | 0.06-22.09 | - |  | 0.73 | 0.02-23.07 |
| Mildly frail | 12.14 | 0.35-411.66 | - |  | 9.13 | 0.21-394.87 |
| Moderately frail | 6.22 | 0.22-175.33 | - |  | 2.51 | 0.05-113.33 |
| Severely frail | - |  |  |  | - |  |
| ***HGS*** | 0.88 | 0.78-0.99* | - |  | 0.87 | 0.71-1.06 |

CFS, clinical frailty scale score; HGS, hand grip strength

**S4b Table: Multivariable Model of the Correlates of Cognitive Impairment (Female Participants)**

| **Female** | **Overall** | | **Age <65** | | **Age** $\boldsymbol{\geq}$**65** | |
| --- | --- | --- | --- | --- | --- | --- |
|  | **OR** | **95% CI** | **OR** | **95% CI** | **OR** | **95% CI** |
| ***Age (years)*** | 1.06 | 1.01-1.11 | 1.02 | 0.84-1.23 | 1.06 | 0.99-1.13 |
| ***Highest level of education*** |  |  |  |  |  |  |
| None | Ref |  | Ref |  | Ref |  |
| Primary | 0.57 | 0.24-1.36 | 0.26 | 0.02-3.06 | 0.54 | 0.19-1.52 |
| Secondary | 0.43 | 0.08-2.35 | 0.11 | 0.01-5.56 | 1.64 | 0.13-20.55 |
| Tertiary | - |  | - |  | - |  |
| ***Marital status*** |  |  |  |  |  |  |
| Currently married | Ref |  | Ref |  | Ref |  |
| Widow/widower | 2.67 | 0.37-19.05 | - |  | 3.96 | 0.35-43.94 |
| Separated/single | 1.88 | 0.20-17.72 | - |  | 1.71 | 0.09-30.70 |
| ***Living situation*** |  |  |  |  |  |  |
| Lives alone | Ref |  | Ref |  | Ref |  |
| Spouse and children | 0.96 | 0.08-11.16 | - |  | 1.84 | 0.06-52.62 |
| Spouse | 2.01 | 0.21-19.28 | - |  | 2.12 | 0.11-40.69 |
| Extended family | 1.04 | 0.35-3.03 | - |  | 1.27 | 0.38-4.17 |
| Children | 0.56 | 0.22-1.40 | - |  | 0.58 | 0.21-1.61 |
| ***Body Mass Index*** |  |  |  |  |  |  |
| Underweight | Ref |  | Ref |  | Ref |  |
| Normal | 1.11 | 0.42-2.92 | - |  | 0.68 | 0.21-2.16 |
| Overweight | 0.39 | 0.11-1.33 | - |  | 0.32 | 0.08-1.30 |
| Obese | 0.33 | 0.09-1.19 | - |  | 0.31 | 0.07-1.29 |
| ***CFS*** |  |  |  |  |  |  |
| Very fit | 5.88 | 0.91-37.81 | 0.93 | 0.05-15.28 | 55.79 | 2.65-1173.84* |
| Well | Ref |  | Ref |  | Ref |  |
| Managing well | 3.96 | 1.05-14.85 | 1.31 | 0.13-12.97 | 9.01 | 1.05-76.99* |
| Vulnerable | 3.51 | 0.77-15.97 | - |  | 7.74 | 0.83-71.80 |
| Mildly frail | 2.01 | 0.22-17.70 | - |  | 4.21 | 0.24-72.27 |
| Moderately frail | 6.88 | 0.35-133.51 | - |  | 13.76 | 0.45-416.92 |
| Severely frail | - |  | - |  |  |  |
| ***HGS*** | 0.91 | 0.84-0.99* | 0.95 | 0.79-1.14 | 0.88 | 0.80-0.98* |

CFS, clinical frailty scale score; HGS, hand grip strength
